# Factors influencing type 2 diabetes self-management practices in rural Bangladesh: a qualitative investigation

**DOI:** 10.3389/fpubh.2024.1508204

**Published:** 2025-01-15

**Authors:** Hasina Akhter Chowdhury, Baki Billah, Shamia Akther Dipa, Ashraful Kabir, A. K. M. Fazlur Rahman, Liaquat Ali, Anju E. Joham, Cheryce L. Harrison

**Affiliations:** ^1^Department of Epidemiology and Preventive Medicine, School of Public Health and Preventive Medicine, Faculty of Medicine, Nursing & Health Sciences, Monash University, Melbourne, VIC, Australia; ^2^Centre for Injury Prevention and Research, Bangladesh (CIPRB), Dhaka, Bangladesh; ^3^Centre for Qualitative Research, Dhaka, Bangladesh; ^4^Pothikrit Institute of Health Studies (PIHS), Dhaka, Bangladesh; ^5^Monash Centre for Health Research and Implementation (MCHRI), Faculty of Medicine Nursing and Health Sciences, Monash University, Clayton, VIC, Australia; ^6^Department of Diabetes, Monash University, Melbourne, VIC, Australia

**Keywords:** barriers, facilitators, diabetes self-management practices, type 2 diabetes mellitus, rural Bangladesh, qualitative study

## Abstract

**Introduction:**

Type 2 diabetes mellitus (T2DM) is a prevalent, chronic health condition of global significance, with low- and middle-income countries (LMICs) disproportionately affected. Diabetes self-management practices (DSMP) are the gold-standard treatment approach, yet uptake remains challenge in LMICs.

**Purpose of the study:**

This study aimed to explore the barriers to and facilitators of DSMP and preferences for intervention design and delivery in Bangladesh, an LMIC, with prevalent T2DM.

**Methods:**

Sixteen qualitative focus group discussions (FGDs) with adults with T2DM and their caregivers were conducted in rural Bangladesh to explore preferences, barriers, and facilitators for community DSMP-related intervention programs. Data were thematically analyzed using a deductive theoretical domains framework (TDF) underpinned by the socio-ecological model.

**Results:**

Overall, 117 participants (*n* = 58 with T2DM and *n* = 59 caregivers) were included in the analysis. Five overarching themes were identified, including (i) implementation of DSMP, (ii) community spirit and interconnectedness, (iii) environmental influences, (iv) healthcare professionals’ role in DSMP, and (v) government support. Key barriers to DSMP identified for T2DM patients include knowledge implementation gaps, cultural practices, limited resources, and financial constraints. Facilitators include motivation, support from family and peers, and religious practices. Rural Bangladeshis prefer programs delivered at community clinics, viewing them as reliable, culturally appropriate central ‘hubs’ to assemble.

**Conclusion:**

Barriers to and facilitators of DSMP were identified, and preferences for intervention design and delivery for implementing DSMP were explored. The findings provide a foundation for the critical need to implement programs that improve DSMP in Bangladesh, with the potential to translate to other LMIC settings.

## Introduction

Type 2 diabetes mellitus (T2DM) is a prevalent, chronic metabolic health condition of global significance, affecting 537 million adults aged 20–79 years worldwide (1). Low-and middle-income countries (LMICs) are disproportionately affected with ~80% of total T2DM cases, yet with fewer healthcare resources than high-income countries (HICs) (2). Within LMICs, those residing in rural areas have an increased risk of adverse health outcomes associated with T2DM compared with their urban counterparts (3, 4). Contributing factors are multifaceted, including higher proportions of geographical and financial disadvantages, limited access to healthcare, delayed journey to diagnosis, and a higher prevalence of individual risk factors, including sedentary lifestyle, and excess weight (5, 6). Addressing the burden of disease associated with T2DM in LMICs requires an interdisciplinary approach, encompassing individual, community, and health systems strategies (7, 8).

Diabetes self-management practices (DSMP) are currently recognized as the gold-standard treatment approach for improving glycemic control (9). They emphasize patient empowerment through education, lifestyle modification, and regular self-monitoring (10–12). Yet, uptake of DSMP in LMICs remains a challenge due to: (i) individual barriers [i.e., socioeconomic disadvantages, differing cultural practices, and beliefs (13–15), linguistic barriers (16, 17)during clinical encounters (18–20)]; (ii) environmental barriers (i.e., geographical impediments, resource and infrastructure constraints limiting access to healthy food, exercise facilities, medication, and transportation); and (iii) health system barriers (i.e., healthcare provider shortages, and resource limitations) (21). Therefore, tailoring DSMP to accommodate these barriers is crucial, requiring context-specific nuanced approaches (10, 13). Despite this, little evidence exists for facilitators of DSMP in LMICs, particularly in rural settings where there are maximum disadvantages (22–24), including higher morbidity and mortality rates (2, 22, 25). Additionally, up to 80% of those with T2DM exhibit poor adherence to blood glucose monitoring (26, 27).

Bangladesh is an LMIC with ~61.8% of its population residing rurally and an escalating T2DM prevalence projected to double from 14.2% (28), within the next two decades (29). Therefore, Bangladesh is an opportune setting in which to explore individual and community perspectives of DSMP to inform strategies to increase uptake and adherence. The aim of this study was to explore the barriers and facilitators of DSMP perceived by people with T2DM and their caregivers in Bangladesh and explore preferences for future intervention design and delivery to improve adherence and health outcomes in Bangladesh, with potential for translation to other LMICs settings.

## Materials and methods

### Study design and population

Detailed study methodology has been previously published and is presented here in brief (19). The study adopts an exploratory qualitative design underpinned by Bronfenbrenner’s ecological system theory (30) and Whittemore et al.’s (31) conceptual framework based on the social-ecological model (19, 32–34). Data collection methods included focus group discussions (FGDs) to enable the exploration of various aspects of facilitators and barriers to T2DM self-management practices. There was a special focus on social context through gathering perspectives on viewpoints, thoughts, expectations, and suggestions (35, 36) from people with T2DM and their caregivers in rural Bangladesh. Furthermore, FGDs investigated preferences, barriers, and facilitators for community-based DSMP intervention programs to meet the unique needs and circumstances of the rural population.

### Study setting

**Figure 1:**
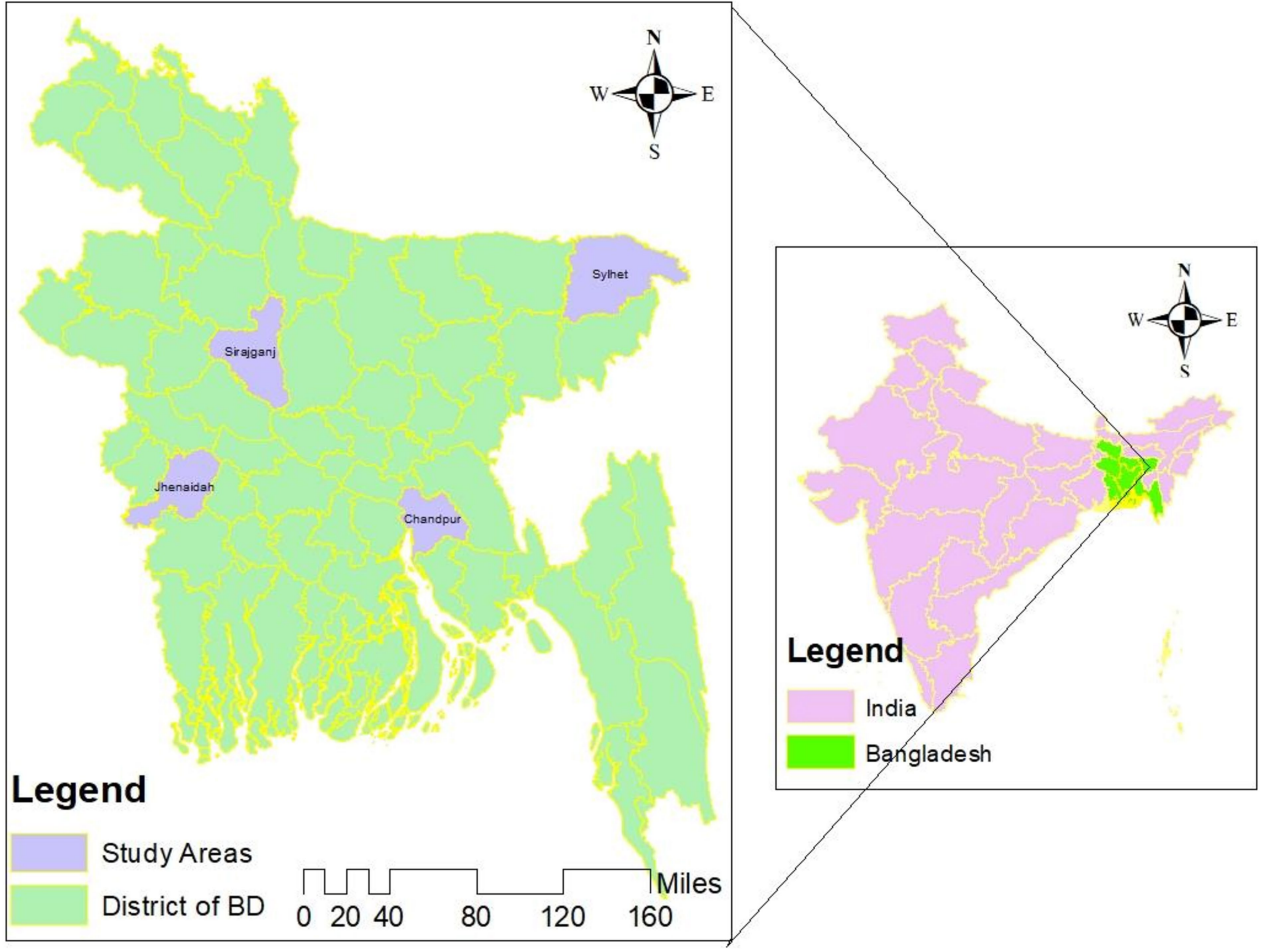
Study site map (19).

The study has been conducted in Bangladesh, a lower-middle-income country in the South Asian region. Bangladesh is geographically divided into eight administrative regions, known as divisions (the first layer), which are further subdivided into 64 districts (the second layer). Each district has several upazilas (the third layer), and each upazila comprises several union parishads (the lowest layer). Each union Paris had is further divided into approximately ~15 to 20 villages (37). This research was implemented in four districts: Jhenaidah et al. encompassing four Upazilas and eight villages (Figure 1). The selected villages were geographically dispersed to ensure a diverse representation of the population. The sampling process has been detailed previously (19).

### Sampling strategy and study participants

Using purposive sampling (38, 39), two community champions, who were local social workers and well-connected with the community, facilitated engagement with potential participants in common settings (i.e., mosques, community centers, and local village markets or tea stalls). They initiated this during religious or community gatherings leveraging their trustable community relationships to explain the study’s purpose and encourage participation.

Participants were recruited in dyads, which included an immediate caregiver to enable the exploration of relationships and roles in T2DM management. Eligibility for participants with T2DM included adults who were (i) aged ≥18 years, (ii) diagnosed with T2DM at least 1 year before recruitment, and (iii) under the active care of a registered physician. Eligibility for caregivers of those with T2DM included (i) a spouse or immediate family member residing with the participant with T2DM, (ii) a provider of support and care to the participant with T2DM at home for at least 1 year prior to data collection (32, 40, 41). Exclusion criteria for all participants included those diagnosed with cognitive or mental impairment or other significant illnesses preventing participation.

### Content of the focus group discussion

Focus group discussion (FGD) interview guide (attached in Appendix 1) included key topic areas of knowledge, motivation, and routines toward DSMP, financial and time constraints, emotional health and well-being, self-monitoring, healthy eating, physical activity, and overall facilitators, barriers, and expectations for continuity of diabetes care. The interview guide also included the key topic areas of expectations for developing a community-based practice approach to implementing DSMP (e.g., the participants’ preferences for the place, mode of intervention delivery, intervention format, duration of intervention, preferred provider, and financial issues). Interview guides were piloted (sample size *n* = 4, data not presented) to ensure cultural sensitivity and acceptability; they were iteratively revised as required. Widely framed and open-ended questions provided ample opportunities for the study participants to share their personal experiences that facilitate and impede their habits and expectations toward DSMP. Participants were also encouraged to shape their own views and thoughts and contribute any additional information relevant to the topic.

### Data collection procedure

The focus group discussions were conducted between July and October 2023 and consisted of eight to 10 participants to facilitate in-depth and manageable interactions. Three interviewers (HAC, ZI, RR) conducted FGDs in Bangla, the native language of both the interviewers and participants. Each FGD was conducted at a mutually convenient location and time, averaging 45–60 min. Discussions were conducted until data saturation was reached, which was indicated when no further information, dimensions, or ideas emerged from the interviews, as outlined by Guest et al. (42, 43). Interviews were audio-recorded and transcribed verbatim by an independent transcribing service (at the Center for Qualitative Research, Dhaka, Bangladesh) and, then translated into English by the first author (HAC) and reviewed by another author (AK). Participant details were deidentified for anonymity.

### Theoretical domains framework and data analysis

This study was guided the Theoretical Domains Framework (TDF), which builds on the Bronfenbrenner’s social-ecological system theory (30) and Whittemore et al.’s conceptual framework (31), rooted in the social-ecological model (SEM), which is widely applied in health services research, provide a robust basis for understanding and addressing diabetes prevention and management (32). This model suggests that long-term behavior change requires targeting multiple levels of influence for a program to succeed. Influence levels are categorized into intrapersonal factors, interpersonal factors, institutional factors, community factors, and public policy levels (30, 31, 33, 34, 44). Intrapersonal factors assess individuals’ ability to change their behaviors to optimize health in diabetes self-management practices, which are influenced by knowledge, attitudes, self-efficacy, and self-confidence (45, 46). A patient’s interpersonal relationships within a societal context, such as family, caregivers, friends, neighbors, colleagues, and healthcare service providers, positively influence an individual’s DSMP (33, 47–49). Social support consists of interpersonal transactions and includes companionship and emotional, tangible, and informational care (37). Institutional influences on individuals include work, school or college, and religious surroundings. Institutional influences provided a structural framework to promote healthy behaviors and prevention activities for diabetes-related comorbidities to a large group, thus forming social supports that further facilitate healthy lifestyle adoption (33). Their prescribed geographical location determines an individual’s community factor. Evidence indicates that the characteristics of the neighborhood and communities affect individuals’ health behavior (31, 46, 50). Further, public policy influences individuals’ health behaviors through the execution of laws and policies by local and national authorities. This model is considered an appropriate framework to explain the dynamic interplay of multiple factors that acknowledge influencing causes as either facilitators, barriers, or expectations to T2DM self-management practices in rural Bangladesh (19).

Transcripts were independently coded by two researchers (HAC and SAD) using the NVivo 12 software (Nvivo [Version 12], QSR International Pty Ltd., Melbourne, Australia [2018]). Data were searched for concepts in relation to research questions using thematic deductive analysis according to the ‘15-point checklist of criteria for efficient thematic analysis’ by Braun and Clark (43). The process involved: (a) establishing a set of *a priori* codes derived from literature and the collective expertise of the research team, (b) line-by-line coding based on the predefined set of codes, (c) initiating coding using both a priori and posteriori codes, incorporating insights from the transcripts, (d) reviewing and organizing all coded data to create a thematic matrix, (e) integrating a priori and posteriori codes into final themes and sub-themes, and (f) presenting the findings under these identified themes. Themes and subthemes were derived by organizing and clustering related codes into coherent groups, followed by iterative discussions and refinement based on emerging patterns and insights from the FGDs and participant feedback. Any disagreements were discussed until a consensus was reached (51). The research findings were presented in accordance with the ‘Consolidated Criteria for Reporting Qualitative Studies: 32-Item Checklist’ (36). All quantitative data were presented as mean (± standard deviation [SD]) unless otherwise stated.

## Results

Overall, we recruited 117 participants (*n* = 58 with T2DM, and *n* = 59 caregivers) and conducted 16 FGDs (*n* = 8 each for T2DM and caregiver participants) ranging in size between eight and 10 participants. The mean age (in years) for people with T2DM was 54.0 (±12.4), with the group comprising 26 males and 32 females. The majority of participants had been living with T2DM for over 5 years. Caregivers’ mean age (in years) was 39.3 (±15). Full participant characteristics are provided in Table 1.

**Table 1:** Socio-demographic characteristics of participants (participants: *n* = 117; FGD: *n* = 16).

Variables	Study sites				
	Jhenaidah	Sylhet	Chandpur	Sirajganj	Combined
People with T2DM (*n* = 58)					
Age in years (mean ± SD)	54.0 ± 11.5	56.8 ± 10.0	57.3 ± 11.0	47.7 ± 15.1	54.0 ± 12.4
Gender					
Male	6	7	6	7	26
Female	9	6	10	7	32
Religious identity					
Muslim	15	13	16	12	56
Hindu	–	–	–	2	2
Education					
No formal education	1	3	1	3	8
Primary (I - V grade)	6	3	4	3	16
Secondary (VI - X grade)	7	3	8	4	22
Higher (> X grade)	1	4	3	4	12
Occupation					
Service	2	–	1	4	7
Housewife	8	6	10	4	28
Business	1	4	3	4	12
Farmer	2	1	–	1	4
Retired	2	2	2	1	7
Duration of diabetes					
≤5 years	7	6	6	4	23
6 to 10 years	5	3	5	7	20
>10 years	3	4	5	3	15
Caregiver (*n* = 59)					
Age in years (mean ± SD)	38.0 ± 15.2	45.6 ± 19.4	32.3 ± 10.5	38.5 ± 9.5	39.3 ± 15
Gender					
Male	2	6	3	5	16
Female	11	7	15	10	43
Religious identity					
Muslim	13	13	18	11	55
Hindu	–	–	–	4	4
Education					
No formal education	4	1	–	2	7
Primary (I - V grade)	6	7	5	6	24
Secondary (VI - X grade)	1	2	10	6	19
Higher (> X grade)	2	3	3	1	9
Occupation					
Service	–	1	–	–	1
Housewife	9	5	14	10	38
Student	2	1	3	1	7
Business	-	3	–	–	3
Farmer	1	2	1	4	8
Retired	1	1	–	–	2

Five overarching themes (Table 2) according to the TDF were identified and are presented below, they include (i) implementation of DSMP (ii) community spirit and interconnectedness, (iii) environmental influences (iv) healthcare professional role in DSMP and (v) government support.

**Table 2:** Themes and sub-themes mapped to self-management practices among people with type 2 diabetes and their caregivers in rural Bangladesh.

Themes and sub-themes	Barrier	Facilitator	TDF domain
**Theme 1: Implementation of DSMP** Sub-themes			Intrapersonal factors
Motivation		**+**	
Implementation barriers	**+**		
Cultural and religious values		**+**	
Financial constraints	**+**		
**Theme 2: Community spirit and interconnectedness** Subthemes			Interpersonal factors
Social support from close-knit	**+**	**+**	
Support from broader social networks (e.g. neighbors/ friends/community people)		**+**	
**Theme 3: Environmental influences** Subthemes			Community factors
Availability and accessibility of community infrastructure and resources	**+**		
Community influences/ social stigma	**+**		
**Theme 4: Healthcare service professional role in DSMP** Sub-themes			Institutional/systematic factors
Doctor-patient relationships	**+**	**+**	
Lack of accessibility/resourcing in the local hospital care	**+**		
**Theme 5: Governmental support**	**+**		Government/Public funding policy factors

The + corresponds to the sub-themes (whether they were barriers or facilitator); TDF, Theoretical Domains Framework.

### Theme 1: Implementation of DSMP

Four subthemes were identified that underpin the theme of DSMP implementation including motivation, implementation barriers, cultural and religious values, and financial constraints.

Participants with T2DM and their caregivers stated that motivation toward a healthy lifestyle, including regular exercise, selecting healthier dietary options, regular glucose monitoring, and medication compliance, was critical for sustained DSMP adherence.

*“Diabetes is a lifelong disease. If I am unwell, I cannot work. So, to stay healthy, I must eat healthy foods regularly, take medicine regularly, and be pleasant to my family, I must strive to manage my diabetes.” (FGD5, T2DM, R6, Female).*

Participants with T2DM also cited their family as a significant source of extrinsic motivation to ensure optimum health and avoid financial burdens on family members.

*“[I want to ensure] improved health outcomes, being healthy for family members, reducing the cost of diabetes care and not to be a burden on [my] children.” (FGD5, T2DM, R2, Female).*

While participants often demonstrated knowledge of the general aspects of a healthy diet or healthier choices and motivation facilitated ongoing behavior change, cultural traditions related to meal preparation frequently presented as a primary barrier to implementing changes. Participants often perceived traditional Bangladeshi diets as difficult to modify, particularly substituting or omitting foods high in complex carbohydrates (e.g., rice) that influence blood glucose levels. This, in part, may be owing to financial constraints related to purchasing alternatives at a higher price point.

*“We prefer to consume more rice and potatoes because these are available and convenient for us. Our rural community, has a tradition of using excessive oil and spices during cooking and males influence household diet decisions*.” (*FGD7, T2DM, R5, Female*).

Further, many participants reported confusion in changing their diet to improve glycemic control, leading to increased reliance on medication to manage blood glucose levels.

*“I was not aware that drinking soft drinks… for example, Coca-Cola/RC water, could raise my sugar levels. Now, my sugar level is 17.6, and I am managing it with insulin and medication.” (FGD3, T2DM, R5, Male).*

In addition to dietary challenges, participants faced physical health limitations that affected their ability to engage in essential DSMP activities. Poor eyesight made it difficult to read medication labels and monitor blood sugar levels accurately, while foot soreness restricted mobility and reduced opportunities for physical exercise. Time constraints and competing responsibilities were also noted as significant barriers, particularly for female participants. Many women expressed being overburdened with household tasks and caregiving responsibilities, leaving them too fatigued to incorporate additional activities, such as walking or exercising, into their daily routines. These challenges highlight the interplay of individual, physical, and socio-cultural factors in shaping DSMP behaviors.

*“My time is spent looking after my family members and [I] have to do all household work from morning to evening. I do not have the energy to go for an additional walk…”(FGD9, T2DM, R1, Female).*

Several participants perceived an interconnection between cultural beliefs and DSMP, noting that beliefs in alternative medicines influenced their initiation and adherence to prescribed medications.

*“I prepare a daily powder using sajina (Moringa) leaves, which has significantly improved my glycaemic status and overall health. Previously, I experienced some vision issues, but now my eyesight has greatly improved.” (FGD7, T2DM, R4, Male).*

Further, most participants reported that their religious beliefs were deeply entrenched and personal, positively influencing their DSMP, with prayer and faith providing a sense of control over their health and improving glycemic control. This also encouraged regular physical activity by attending mosques and promoting discipline and social interaction in rural communities.

*“My wife prays regularly (at least five times a day). Performing prayer is a kind of exercise and brings her mental tranquillity.” (FGD17, Caregiver, R2, Male).*

Financial constraints presented a barrier to DSMP, contributing to reduced access to resources and the inability to meet healthcare costs related to medical consultations, diagnostic tests, buying blood glucose monitoring devices, medications, and ongoing consumables. As a result, some participants reported refraining from using health services.

*“We are poor people….But every month, a significant amount of money is required for numerous tests, which are not performed due to financial constraints. Money is also needed for doctor visits and medicines. Then, we either avoid going to the clinic or skip the tests.” (FGD11, T2DM, R1, Male).*

### Theme 2: Community spirit and interconnectedness

The theme of community spirit and interconnectedness is underpinned by two subthemes including social support from close-knit relationships (e.g., spouse, children, and in-laws) and broader social networks (e.g., neighbors, friends, and community members).

The presence of social support from family members (e.g., spouse, son/daughter, and daughters-in-law/sons-in-law), appeared to have an inverse relationship with the ability to engage in and maintain, DSMP effectively. This was particularly pronounced in the case of female participants, who were more likely to report receiving less support, challenging their ability to engage in healthy self-management practices. In contrast, participants who reported receiving ongoing support were likelier to have increased motivation and ability to implement DSMP. In addition, caregivers enhance well-being by facilitating self-management. Their support fosters an environment that facilitates effective DSMP by addressing medical, emotional, and logistical needs, ultimately leading to better health outcomes and an improved quality of life for the people with T2DM.

*“No, my family does not provide any such assistance. My husband died five years ago. I lack healthy meals and medicines at home. My children do not accompany me to doctor appointments.”(FGD18, T2DM, R5, Female).*

*“I am privileged to have a supportive family who consistently encourages me to maintain an active lifestyle, adhere to medication, and follow a healthy diet. Additionally, my friendships with people with T2DM are beneficial; we motivate and support each other by sharing experiences in managing diabetes.”(FGD3, T2DM, R4, Male)*.

*“I accompany my husband to the hospital, prepare his meals, remind him to take his medicine, and support him daily., I’ve been involved in everything for seven years, through good and bad times.” (FGD10, Caregiver, R2, Female).*

The supportive roles of neighbors, peers, or community members in rural settings were essential for providing emotional support, advice, and lived experience of diabetes management strategies and practical support, including help buying medications.

*“Although no one helps us financially, sometimes some of our community people give us suggestions like which food my mother needs to consume or which food she needs to avoid.” (FGD4, Caregiver R5, Female).*

### Theme 3: Environmental influences

This theme includes two sub-themes: availability and accessibility of community infrastructure and resources, and community influences/social stigma.

Most participants reported that barriers to DSMP included a lack of healthy food options in markets, lack of exercise facilities, busy roads or narrow village paths, and related to safety issues, such as crime or traffic hazards that hinder walking in the local community setting.

*“We want to go for a walk on the streets every morning to control our diabetes. But many vehicles use this road. The doctor asked us to walk fast, but it is not possible on this road. Similarly, we cannot walk on the village road because it is too narrow. In addition, there are no separate facilities for walking for our female family members. So, this is a big problem!!” (FGD7, T2DM, R1, Male).*

Participants reported that social stigma negatively impacts people with T2DM. Most of the participants shared that the community perceives T2DM as a curse, often blaming those affected and fostering in them feelings of personal failure or guilt regarding their health conditions. Moreover, many female participants described receiving verbal abuse due to societal norms and perceptions during physical exercise.

*“Many community people say many things…, for example, ‘Allah has cursed her with so much sickness that she went out for a walk so early in the morning without sleeping’.”(FGD7, T2DM, R5, Female).*

*“When my mother goes for a walk, community people verbally harass her and make derogatory comments behind her back.” (FGD6, Caregiver, R3, Female).*

### Theme 4: Healthcare professionals’ role in DSMP

This theme includes two sub-themes: doctor-patient relationships and lack of accessibility/resourcing in local hospital care. Participants cited their doctor-patient relationship as significantly influencing DSMP. They perceived doctors and nurses as generally uncooperative, prioritizing quick transactions over sincere support due to high patient volumes. These accounts may underscore systemic issues, including provider and medicine shortages, leading to patient dissatisfaction and reluctance to seek further medical treatment.

*“I go to the specialised hospital, where the doctor sits far away and prescribes medicine, and insulin after listening to the patient. He advises, ‘Walk for two hours and control your diet’ That’s it! This distance makes having a friendly, supportive relationship with the doctor difficult.” (FGD9, T2DM, R8, Male).*

Some participants noted that doctors are crucial sources of information on critical aspects of DSMP. Participants expressed that information on healthy eating, physical exercise, and foot care was also beneficial; this information was utilized by people with T2DM in their self-management and shared with family members, fostering a supportive home environment for DSMP.

*“Doctors and health staff provide information on the importance, and continuity of medicine, foot care, physical exercise, and proper diet to manage diabetes. They advise us to maintain the diabetes management guidelines.” (FGD3, T2DM, R5, Male).*

Further, participants in rural areas faced linguistic barriers as doctors primarily communicated in English or used complex medical terms, leading to difficulty in understanding instructions. Consequently, many people with T2DM felt the healthcare system did not meet their personal needs in managing diabetes.

*“We often struggle to understand the words doctors use or make sense of complicated medical terms.” (FGD13, T2DM, R2, Female).*

Participants perceived community clinic locations and services as convenient for diabetes care. Yet, the majority noted anecdotally a significant decline in the availability of diabetes medication at these clinics over recent years, with medications largely unavailable, affecting their ongoing treatment. Further, participants faced critical gaps in diabetes care, affecting both testing accuracy and treatment consistency; long distances, transport costs, and considerable waiting times posed a significant risk to their health.

*“The community clinic near us…..only provides check-ups but lacks further diabetes treatment. I visit for diabetes testing, which costs 30 TK, but the glucometer often gives inaccurate results or does not work properly.” (FGD7, T2DM, R1, Male).*

*“The local hospital is very far away from home and 100 TK is needed for transportation; …it’s difficult to take my father to the hospital.” (FGD10, Caregiver, R2, Female).*

### Theme 5: Government support

Participants expressed concerns about the lack of government support for people with T2DM in rural areas, noting that despite various allowances offered for other needs, there is a significant gap in providing essential medical equipment. This shortfall forces many patients to incur additional financial burdens or forgo necessary treatments.

*“The government provides various allowances to many people, such as Old Age Allowance, Widow* Allowance*, Rice Oil Card, and so on; however, they do not supply sufficient machines for diabetic patients.” (FGD5, T2DM, R4, Female).*

### Participants’ preferences for DSMP program implementation in rural settings

Participants were presented with preferences for DSMP intervention design, including setting, delivery mode, program format, content, and facilitator type, as well as barriers to and enablers of engagement. Overall, participants preferred programs delivered at community clinics as they considered them reliable, culturally appropriate central ‘hubs’ to assemble. Alternatively, home-based services were chosen based on safety and convenience concerns, particularly by women or those with mobility issues. Group-based attendance was favored for its potential to enhance learning through shared experiences, with a strong preference for resources with illustrations (rather than only written material) and involving family members for additional support in implementing the advice received. Participants emphasized their preference for programs delivered by those with a medical background (e.g., specialized doctors). However, knowledgeable professionals were also considered trustworthy. Financial constraints were a significant concern, with many participants advocating for free or subsidized programs to alleviate the economic burden of diabetes management. These details are included in Table 3.

**Table 3:** Expectations from people with T2DM and their caregivers for attending any DSMP-related educational program/service and exemplar quotes.

Topic	Preferences of participants regarding the DSMP educational program	Exemplar quotes
Where would you prefer to attend any DSMP-related educational program/service?	**Community clinics:** Participants highly preferred these due to the perceived reliability and the ability to maintain cultural practices. **Home-based services:** Valued for their convenience, especially for women or those who have mobility issues. **Schools and mosques:** Seen as convenient central points but pose cultural challenges for women.	*Our community clinic would be better placed to conduct the program; everyone will get the services properly if it is provided in the clinic. [FGD15, T2DM, R1, Male]* *As women, we feel discomfort going outside. Therefore, it would be best for us if they came to our house once a month, served us, and gave us advice and medicine. [FGD13, T2DM, R3, Female]* *It would be best to choose a quiet place (*e.g.*, mosque) suitable for many people. Now everyone has diabetes at home. [FGD4, Caregiver, R3, Female]*
How would you prefer to attend this service?		
Group or individual attendance?	**Group-based:** Most participants preferred a group-based setup for attending the service. They believe it enhances learning and allows for shared experiences.	*It will be great if you arrange the program in a group format. If the service is made available to a group of people, all messages are easily shared with everyone. We can learn everything about diabetes and how to manage it. [FGD5, T2DM, R4, Male]*
With your family members or not?	**With family members:** Participants are open to involving family members. They believe family members can provide support and reminders about managing diabetes.	*Family members must be there for support and awareness. [FGD4, Caregiver, R2, Male]*
Delivered by who?	**Experts and specialists:** There is a strong preference for services to be delivered by experts, particularly doctors who are experienced in diabetes care. **Volunteers and knowledgeable individuals:** In some cases, participants mentioned that knowledgeable volunteers or individuals within the community could also deliver the services effectively.	*It would be better if specialist doctors provided the service. [FGD11, T2DM, R4, Male]*
Educational materials	**Pictorial materials:** A significant number of participants emphasized the importance of pictorial educational materials over text. This is especially important for those who are illiterate or have difficulty understanding written instructions. **Text and pictures:** Some suggested that combining text with pictures would be beneficial for better understanding.	*It would be better if you put a picture on it. If it is written, an illiterate person will not understand it. Everyone can understand it if you give a picture. Therefore, having a book with explanations and pictures would be incredibly helpful for you. [FGD18, T2DM, R2, Male]* *Making a concise manual with pictures helps understand what should be done, especially for those who cannot read. [FGD16, Caregiver, R3, Female]*
With ongoing support via regular phone calls or home visits	**Phone calls and home visits:** Regular support through phone calls, or home visits, is appreciated and considered beneficial for continuous diabetes care. **Community Clinics:** Participants expressed a desire to receive support from nearby community clinics.	*It will be good via phone calls or home visits. [FGD17, Caregiver, R4, Male]*
Who would motivate you to attend?	**Family and relatives:** Family members play a crucial role in motivating participants to attend. **Community and service providers:** Announcements from service providers or trusted community members can also encourage attendance.	*My family members will push me to attend the health program. [FGD3, T2DM, R1, Male]* *I have to tell my husband that going there is important. I have to insist on him attending the program rather than working. [FGD8, Caregiver, R1, Female]*
Opinions about financial matters	**Financial support:** There is a need for financial assistance, both in terms of the cost of medicines, free glucometers, and possibly other supportive measures. **Free services:** Some participants strongly advocate for free services to alleviate the financial burden on patients.	*Whoever has the machine can do the diabetic test himself. It is better to give a machine to those who do not have it. [FGD18, T2DM, R1, Male]*
How intensely would you like to be supported?		
	**Monthly programs:** Participants preferred programs where they could get their medicine monthly. This ensures that they have a continuous supply and can manage their condition effectively. **Seasonal adjustments:** Some suggested that winter is a better season for organising programs due to better road conditions and higher participant turnout. **Timing:** Programs should ideally be scheduled between 9 am and 12 pm, as this is a convenient time for most participants, and during times that do not conflict with work schedules (e.g., Friday).	*Regular check-ups and regular medicine are needed for diabetes management. So, if the services are provided every month, then it would be better for us. [FGD13, T2DM, R3, Female]* *If the program is arranged in winter. That will be better. Roads are good in winter. [FGD18, T2DM, R1, Male]* *Programs should ideally be scheduled between 9 am and 12 pm. Friday is convenient time for most participants and does not conflict with work schedules. Then everyone can join this program. [FGD3, T2DM, R1, Male]*
How willing are you to pay for this service?		
	**Affordability concerns:** Participants emphasized their financial constraints, indicating that paying for services is not feasible for many.	*We are poor people. Free service will be good for us. [FGD15, T2DM, R1, Female]*
	**General preference for free services:** Many participants strongly prefer free services due to their economic conditions.	*We are poor people. We want free services. [FGD15, T2DM, R3, Female]* *If the service is provided completely free it would be much better for us. [FGD14, Caregivers, R3, Female]*
	**Conditional payment:** Some participants can afford to pay but believe those who cannot should receive free services.	*I can afford some money but for those who are poor and cannot afford the expenses, it should be free for them. [FGD3, T2DM, R1, Male]*
	**Economic hardships:** There is a lack of financial resources to pay for necessary medical services.	*We have lack of financial resources to pay for government services. So, we want it without any charge. [FGD5, T2DM, R4, Male]*

## Discussion

By adopting a socio-ecological pragmatic approach, this qualitative study provides valuable insights into the complex interplay of factors influencing DSMP. It identifies five TDF domains that act as barriers or enablers for DSMP, in addition to intervention program preferences, in rural community settings. Overall, the qualitative data identified the following themes: knowledge implementation in DSMP; community spirit, and interconnectedness; environmental influences in DSMP; and healthcare professionals’ role in DSMP; and government support in shaping DSMP. These were the main factors impacting people with T2DM and their caregivers in managing diabetes at home. Further, participants preferred community-based DSMP programs that are group-oriented and led by qualified professionals, focusing on practical resources and affordability. Translating these findings into practice is vital to inform community intervention design and advance knowledge, confidence, and resources.

Motivated individuals with T2DM are likelier to adopt healthy lifestyles, lead an active life, and feel responsible for their health and associated outcomes (52). Here, most participants were motivated; however, they struggled to implement changes due to significant knowledge gaps, cultural traditions, financial constraints, and accessibility issues. In line with previous findings (1, 53, 54), there was a strong preference for carbohydrate-rich foods known to affect glycemic control. Dietary modification to reduce cereals, including rice, presented a significant barrier with cultural and financial considerations. This included social gatherings and festivals where traditional rice dishes are the mainstay, making dietary modifications challenging. Further, we noted gendered differences in household meal preparation. Despite often being the primary preparer of household meals, the females noted the conventional practice of men largely being the decision-makers in household dietary choices; they observed it to be a significant reason for losing control over modifying dietary patterns and substituting foods with healthier options in line with DSMP (54–57).

We observed a direct relationship between barriers in practicing DSMP and social support from family members, and this was more frequently noted in the case of female participants. Consistent with previous findings, a lack of support from family and friends was a barrier to physical exercise (58). Conversely, in this study, participants with ongoing support from family reported enhanced motivation and ability to implement DSMP. As with other studies (1, 59–63), we noted that family support, including, emotional support, motivation, reminders, and companionship, is essential for maintaining DSMP and adhering to recommended practices at home. Beyond the immediate family, support from peers, neighbors, and community members offered emotional and practical assistance. Exploratory studies suggest that sharing knowledge and experiences of DSMP among individuals with T2DM along with the availability of community recreational facilities (64) facilitates physical activity (58, 63, 65, 66). Further, most participants reported a strong connection to their religion, with most regularly attending mosques for prayers; they reported this as enhancing glycemic control and encouraging physical activity, discipline, and social interaction. In this setting, religion aided individuals in leading healthier lifestyles (67), with religious place (e.g., mosque) attendance linked to improved social interactions (68). Religious and cultural practices were deeply seeded within the community, firmly guiding the beliefs and practices of participants, in line with previous research (69–71). Conversely, we discovered that societal views that instilled fear of judgment and harassment led to internalized shame and guilt, which in turn, deterred engagement in health-promoting activities and created barriers to following medical advice. This supports previous findings that social stigma is a significant predictor of lower patient activation levels for self-care in persons with T2DM (72).

While our urban areas benefit from closer proximity to healthcare and reliable transport systems, rural settings face distinct challenges in healthcare access and diabetes management due to their unique geographic, social, economic, and infrastructural characteristics (73). For example, limited transportation options, fewer healthcare facilities, reliance on others for travel, and long distances to clinics with poor road infrastructure hinder access to services (74). Additionally, cultural factors, such as traditional health beliefs and a lack of awareness about modern healthcare practices, can influence attitudes toward seeking and adhering to medical care, further exacerbating the challenges faced in rural areas. In addition, reflecting on the relationship between people with T2DM and their healthcare providers, participants reported a lack of cooperation in communication, resulting in linguistic barriers; this often led to feelings of disrespect, dissatisfaction, and reluctance to seek care. Participants also identified systemic and environmental barriers in local hospitals, including medication shortages, inaccurate testing equipment, prolonged wait times, and the requirement to travel long distances to receive care. This is consistent with a previous study that found irregular medical supplies in health facilities hindered adherence to recommended medications (63). These logistical barriers highlight the need for decentralized healthcare services, such as mobile clinics or telemedicine, to bring care closer to rural populations (75). Financial constraints were also emphasized as a significant barrier to T2DM self-management practices. In Bangladesh, healthcare costs account for 67% of household spending (76), which means people with T2DM often face significant out-of-pocket expenses. Previous research in rural Bangladesh (53) indicated that public hospitals offer free doctor consultations, but patients must pay out-of-pocket costs for laboratory tests, medications, and transportation. This imposes significant financial strain on patients and their families and increases the likelihood of inconsistent engagement with healthcare providers and systems (77) and noncompliance with self-management practices (32) or the belief that self-management efforts are futile (78). Enhancing government support through increased funding, policy implementation, and accountability could improve access to care, support, and resources for people with T2DM (79).

Studies highlight that culturally appropriate diabetes education can enhance glycemic control and improve health behaviors (80), however, little is known about effective educational strategies and methods targeting rural groups with T2DM (81–83). In this study, participants preferred diabetes self-management programs delivered at primary care or community clinics, valued for their reliability and cultural appropriateness. However, some favored home-based services for safety and convenience. Participants preferred group learning, illustrated educational resources, family involvement, delivery by medical professionals, and the importance of free or subsidized programs to ease financial burdens. Previous studies showed that tailored guidance and specific diet and physical exercise information are crucial for goal setting and habit modification and should be regularly provided (38). Given its proximity to patients, easier accessibility, and continuity of care, the primary care setting may be ideal for implementing behavioral counseling interventions to promote patients’ engagement with self-management practices (1).

This TDF that aids in interpreting the data by highlighting the multi-level influences on DSMP in rural communities in Bangladesh. It identifies individual, interpersonal, community, and societal factors that shape behaviors, such as knowledge gaps, cultural traditions, and gendered decision-making in household meal preparation. The TDF emphasizes the importance of social support, particularly from family and community, in facilitating DSMP. Additionally, environmental influences, such as access to resources and community-based interventions, play a critical role. The TDF helps identify these interconnected factors, guiding the design of effective, context-specific interventions for individuals with T2DM.

### Strengths and limitations

The study design was theoretically underpinned by the Theoretical Domains Framework to explore T2DM management and targeted multiple levels of influence (30, 31). To the best of our knowledge, this is the first in-depth formative research, conducted within rural community settings where T2DM is highly prevalent and health services are under significant strain, underscoring the urgent need for model implementation of DSMP. Using a pre-tested interview guide, we provide new insights into the critical factors for self-management of T2DM and community-level preferences for program development. To emphasize the relevance of involving both people with T2DM and their caregivers to address the holistic needs of T2DM self-management practices. We recruited dyads of patients and their caregivers. This approach has not been extensively utilized in previous research. This study is subject to possible limitations. First, the T2DM subjects were from middle-aged to older age groups, and therefore, their responses may not be generalizable to other age groups (such as below 18 years age) with differing priorities and perspectives. Second, we recruited the study participants from rural areas; thus, the results were limited by geographic location. Additionally, the study included people with T2DM who were under the care of a registered physician. This may cause selection bias, as the sample may not represent the broader T2DM population, especially those with limited healthcare access. Despite these limitations, rigorous study methodology enables this to be conceptually transferable. Third, due to the linguistic differences between native Bangla and English, nuances, idiomatic expressions, and cultural contexts in Bangla often lack direct equivalents in English, leading to potential misinterpretations and challenges in accurately conveying participants’ experiences. To address this challenge and ensure an accurate representation of participants’ experiences, we included cultural experts and bilingual translators, in addition to using back-translation, and participant feedback. Fourth, as the study employed a qualitative approach to explore in-depth experiences and perspectives, quantitative data collection was beyond its scope. However, the findings provide a foundational understanding that could inform future mixed-methods research to quantify the impact of the identified barriers and facilitators. Future studies are needed to provide further insight, particularly in urban settings, where providing basic diabetes care (and primary care health care in general) attracts barriers and enablers.

## Conclusion

Type 2 diabetes mellitus subjects in rural Bangladesh are motivated to improve their health by DSMP; however, they face substantial barriers to integrating self-management practices. This includes knowledge and implementation gaps, barriers related to cultural practices particularly affecting rural women, limited availability and accessibility of community and healthcare resources, social stigma, and overall financial constraints. Motivation, family and peer support, and positive religious practices promoting physical activity and social interaction are major facilitators. As preferences by diabetic patients and caregivers, the accessibility and quality of care provided by community clinics as well as home-based services need to be improved with adequate infrastructure, human resources, and essential supplies through public-private initiatives. Thus, the present findings highlight a ‘practical needs’ approach before and during health interventions on DSMP and provide a foundation for future research on implementing DSMP support interventions for rural or disadvantaged populations in similar LMICs.

## Data Availability

The datasets presented in this article are not readily available because the data utilized and examined in this research cannot be publicly disclosed due to ethical restrictions and confidentiality concerns. However, access to the data can be granted upon reasonable request from the corresponding author. Requests to access the datasets should be directed to Baki Billah and email: baki.billah@monash.edu.

## References

[1] Laranjo L Neves AL Costa A Ribeiro RT Couto L Sá AB. Facilitators, barriers and expectations in the self-management of type 2 diabetes—a qualitative study from Portugal. Eur J Gen Pract. (2015) 21:103–10. doi: 10.3109/13814788.2014.1000855, PMID: 25698085

[2] Lam AA Lepe A Wild SH Jackson C. Diabetes comorbidities in low-and middle-income countries: an umbrella review. J Glob Health. (2021) 11:11. doi: 10.7189/jogh.11.04040, PMID: 34386215 PMC8325931

[3] Dugani SB Mielke MM Vella A. Burden and management of type 2 diabetes in rural United States. Diabetes Metab Res Rev. (2021) 37:e3410. doi: 10.1002/dmrr.3410, PMID: 33021052 PMC7990742

[4] Bain LE Adeagbo OA. There is an urgent need for a global rural health research agenda. Pan Afr Med J. (2022) 43:147. doi: 10.11604/pamj.2022.43.147.38189, PMID: 36785680 PMC9922072

[5] Chowdhury HA Paromita P Mayaboti CA Rakhshanda S Rahman FN Abedin M. Assessing service availability and readiness of healthcare facilities to manage diabetes mellitus in Bangladesh: findings from a nationwide survey. PLoS One. (2022) 17:e0263259. doi: 10.1371/journal.pone.0263259, PMID: 35171912 PMC8849622

[6] Wu Y Ding Y Tanaka Y Zhang W. Risk factors contributing to type 2 diabetes and recent advances in the treatment and prevention. Int J Med Sci. (2014) 11:1185–200. doi: 10.7150/ijms.10001, PMID: 25249787 PMC4166864

[7] Flood D Hane J Dunn M Brown SJ Wagenaar BH Rogers EA. Health system interventions for adults with type 2 diabetes in low-and middle-income countries: a systematic review and meta-analysis. PLoS Med. (2020) 17:e1003434. doi: 10.1371/journal.pmed.1003434, PMID: 33180775 PMC7660583

[8] Ong SE Koh JJK Toh SAES Chia KS Balabanova D McKee M. Assessing the influence of health systems on type 2 diabetes mellitus awareness, treatment, adherence, and control: a systematic review. PLoS One. (2018) 13:e0195086. doi: 10.1371/journal.pone.0195086, PMID: 29596495 PMC5875848

[9] Mbride M. Effect of diabetes self-management education utilizing short message service among adults. Grand Canyon University ProQuest Dissertations & Theses. (2022).

[10] Sherifali D Jones H Mullan Y. Diabetes self-management: what are we really talking about? Can J Diabetes. (2013) 37:2–3. doi: 10.1016/j.jcjd.2013.01.003, PMID: 24070741

[11] Ernawati U Wihastuti TA Utami YW. Effectiveness of diabetes self-management education (DSME) in type 2 diabetes mellitus (T2DM) patients: systematic literature review. J Public Health Res. (2021) 10:2240. doi: 10.4081/jphr.2021.2240 33855427 PMC8129774

[12] Koch T Jenkin P Kralik D. Chronic illness self-management: locating the ‘self’. J Adv Nurs. (2004) 48:484–92. doi: 10.1111/j.1365-2648.2004.03237.x 15533086

[13] de J Aweko J Daivadanam M Alvesson H Delobelle P Mayega R. Diabetes self-management in three different income settings: cross-learning of barriers and opportunities. PLoS One. (2019) 14:e0213530. doi: 10.1371/journal.pone.0213530, PMID: 30889215 PMC6424475

[14] Flores G. Culture and the patient-physician relationship: achieving cultural competency in health care. J Pediatr. (2000) 136:14–23. doi: 10.1016/S0022-3476(00)90043-X, PMID: 10636968

[15] Wachtler C Brorsson A Troein M. Meeting and treating cultural difference in primary care: a qualitative interview study. Fam Pract. (2006) 23:111–5. doi: 10.1093/fampra/cmi086, PMID: 16246851

[16] Althubyani AN Gupta S Tang CY Batra M Puvvada RK Higgs P. Barriers and enablers of diabetes self-management strategies among Arabic-speaking immigrants living with type 2 diabetes in high-income Western countries-a systematic review. J Immigr Minor Health. (2024) 26:761–74. doi: 10.1007/s10903-023-01576-0, PMID: 38231345 PMC11289197

[17] Mogre V Johnson NA Tzelepis F Shaw J Paul C. Adherence to self-care behaviours and associated barriers in type 2 diabetes patients of low-and middle-income countries: a systematic review protocol. Syst Rev. (2017) 6:1–6. doi: 10.1186/s13643-017-0436-4, PMID: 28241863 PMC5327551

[18] Masupe T Onagbiye S Puoane T Pilvikki A Alvesson HM Delobelle P. Diabetes self-management: a qualitative study on challenges and solutions from the perspective of south African patients and health care providers. Glob Health Action. (2022) 15:2090098. doi: 10.1080/16549716.2022.2090098, PMID: 35856773 PMC9307110

[19] Chowdhury HA Joham AE Kabir A Rahman AKMF Ali L Harrison CL. Exploring type 2 diabetes self-management practices in rural Bangladesh: facilitators, barriers and expectations—a qualitative study protocol. BMJ Open. (2024) 14:e081385. doi: 10.1136/bmjopen-2023-081385, PMID: 38697759 PMC11086285

[20] Rasmussen B Wynter K Rawson HA Skouteris H Ivory N Brumby SA. Self-management of diabetes and associated comorbidities in rural and remote communities: a scoping review. Aust J Prim Health. (2021) 27:243–54. doi: 10.1071/PY20110 34229829

[21] Dillahunt TR Veinot TC. Getting there: barriers and facilitators to transportation access in underserved communities. ACM Transactions on Computer-Human Interaction (TOCHI). (2018) 25:1–39. doi: 10.1145/3233985

[22] Houle J Lauzier-Jobin F Beaulieu MD Meunier S Coulombe S Côté J. Socioeconomic status and glycemic control in adult patients with type 2 diabetes: a mediation analysis. BMJ Open Diabetes Res Care. (2016) 4:e000184. doi: 10.1136/bmjdrc-2015-000184, PMID: 27239316 PMC4873951

[23] Adu MD Malabu UH Malau-Aduli AEO Malau-Aduli BS. Enablers and barriers to effective diabetes self-management: a multi-national investigation. PLoS One. (2019) 14:e0217771. doi: 10.1371/journal.pone.0217771, PMID: 31166971 PMC6550406

[24] Zhang Z-C du QH Jia HH Li YM Liu YQ Li SB. A qualitative study on inner experience of self-management behavior among elderly patients with type 2 diabetes in rural areas. BMC Public Health. (2024) 24:1456–7. doi: 10.1186/s12889-024-18994-w, PMID: 38822296 PMC11140989

[25] Woodward A Walters K Davies N Nimmons D Protheroe J Chew-Graham CA. Barriers and facilitators of self-management of diabetes amongst people experiencing socioeconomic deprivation: a systematic review and qualitative synthesis. Health Expect. (2024) 27:e14070. doi: 10.1111/hex.14070, PMID: 38751247 PMC11096776

[26] Fottrell E Ahmed N Shaha SK Jennings H Kuddus A Morrison J. Diabetes knowledge and care practices among adults in rural Bangladesh: a cross-sectional survey. BMJ Glob Health. (2018) 3:e000891. doi: 10.1136/bmjgh-2018-000891, PMID: 30057800 PMC6058170

[27] Jennings HM Morrison J Akter K Haghparast-Bidgoli H King C Ahmed N. Care-seeking and managing diabetes in rural Bangladesh: a mixed methods study. BMC Public Health. (2021) 21:1–14. doi: 10.1186/s12889-021-11395-3, PMID: 34294059 PMC8299577

[28] Magliano DJ Boyko EJ Atlas ID. What is diabetes? In: IDF DIABETES ATLAS. 10th ed. International Diabetes Federation (2021) 54–61.

[29] Hossain MB Khan MN Oldroyd JC Rana J Magliago DJ Chowdhury EK. Prevalence of, and risk factors for, diabetes and prediabetes in Bangladesh: evidence from the national survey using a multilevel Poisson regression model with a robust variance. PLOS Global Public Health. (2022) 2:e0000461. doi: 10.1371/journal.pgph.0000461, PMID: 36962350 PMC10021925

[30] Bronfenbrenner U. Ecological systems theory. In Vasta R, editor. Six theories of child development: Revised formulations and current issues. Jessica Kingsley Publishers (1992) p. 187–249.

[31] Whittemore R Melkus GDE Grey M. Applying the social ecological theory to type 2 diabetes prevention and management. J Community Health Nurs. (2004) 21:87–99. doi: 10.1207/s15327655jchn2102_03, PMID: 15123438

[32] Adhikari M Devkota HR Cesuroglu T. Barriers to and facilitators of diabetes self-management practices in Rupandehi. Nepal-multiple stakeholders’ perspective BMC public health. (2021) 21:1–18. doi: 10.1186/s12889-021-11308-4 PMC8243465 34187461

[33] McLeroy KR Bibeau D Steckler A Glanz K. An ecological perspective on health promotion programs. Health Educ Q. (1988) 15:351–77. doi: 10.1177/109019818801500401 3068205

[34] Glanz K. Progress in dietary behavior change. Am J Health Promot. (1999) 14:112–7. doi: 10.4278/0890-1171-14.2.112, PMID: 10724722

[35] Liamputtong P., Research methods in health: Foundations for evidence-based practice. South Melbourne, Vic: Oxford University Press. (2010).

[36] Patton MQ. Qualitative research In: Encyclopedia of statistics in behavioral science. Hoboken: John Wiley & Sons, Ltd. (2005) p. bsa514.

[37] Ford ME Tilley BC McDonald PE. Social support among African-American adults with diabetes, part 2: a review. J Natl Med Assoc. (1998) 90:425–32. PMID: 9685778 PMC2608356

[38] Kabir A Karim N Billah B. Preference and willingness to receive non-communicable disease services from primary healthcare facilities in Bangladesh: a qualitative study. BMC Health Serv Res. (2022) 22:1473. doi: 10.1186/s12913-022-08886-3, PMID: 36463166 PMC9719224

[39] Kabir A Karim MN Billah B. Health system challenges and opportunities in organizing non-communicable diseases services delivery at primary healthcare level in Bangladesh: a qualitative study. Front Public Health. (2022) 10:1015245. doi: 10.3389/fpubh.2022.1015245, PMID: 36438215 PMC9682236

[40] Ahmann E Abraham M Johnson B. Institute for patient-and family-centered care. Emerg Med. (2010) 15:109–11.

[41] Lister J Han L Bellass S Taylor J Alderson SL Doran T. Identifying determinants of diabetes risk and outcomes for people with severe mental illness: a mixed-methods study. Health Services Delivery Res. (2021) 9:1–194. doi: 10.3310/hsdr09100, PMID: 34029027

[42] Guest G Bunce A Johnson L. How many interviews are enough? An experiment with data saturation and variability. Field Methods. (2006) 18:59–82. doi: 10.1177/1525822X05279903

[43] Braun V Clarke V. Using thematic analysis in psychology. Qual Res Psychol. (2006) 3:77–101. doi: 10.1191/1478088706qp063oa

[44] Emmons KM. Health behaviors in a social context. Social epidemiol. (2000) 137:242–65. doi: 10.1093/oso/9780195083316.003.0011, PMID: 39278244

[45] Norris SL Engelgau MM Venkat Narayan K. Effectiveness of self-management training in type 2 diabetes: a systematic review of randomized controlled trials. Diabetes Care. (2001) 24:561–87. doi: 10.2337/diacare.24.3.561, PMID: 11289485

[46] Kaplan GA Everson SA Lynch JW. The contribution of social and behavioral research to an understanding of the distribution of disease: A multilevel approach. The University of Michigan Library. National Academy Press. (2000).

[47] Brown SA Hedges LV. Predicting metabolic control in diabetes: a pilot study using meta-analysis to estimate a linear model. Nurs Res. (1994) 43:362–8. doi: 10.1097/00006199-199411000-00008 7971301

[48] Trief PM Himes CL Orendorff R Weinstock RS. The marital relationship and psychosocial adaptation and glycemic control of individuals with diabetes. Diabetes Care. (2001) 24:1384–9. doi: 10.2337/diacare.24.8.1384, PMID: 11473074

[49] King DK Glasgow RE Toobert DJ Strycker LA Estabrooks PA Osuna D. Self-efficacy, problem solving, and social-environmental support are associated with diabetes self-management behaviors. Diabetes Care. (2010) 33:751–3. doi: 10.2337/dc09-1746, PMID: 20150299 PMC2845021

[50] Sorensen G Emmons K Hunt MK Johnston D. Implications of the results of community intervention trials. Annu Rev Public Health. (1998) 19:379–416. doi: 10.1146/annurev.publhealth.19.1.379 9611625

[51] Goldstein RF Walker RE Teede HJ Harrison CL Boyle JA. The healthy pregnancy service to optimise excess gestational weight gain for women with obesity: a qualitative study of health professionals’ perspectives. J Clin Med. (2020) 9:4073. doi: 10.3390/jcm9124073, PMID: 33348671 PMC7766467

[52] Lakerveld J Palmeira AL van Duinkerken E Whitelock V Peyrot M Nouwen A. Motivation: key to a healthy lifestyle in people with diabetes? Current and emerging knowledge and applications. Diabet Med. (2020) 37:464–72. doi: 10.1111/dme.14228, PMID: 31916283

[53] Ghimire S. Barriers to diet and exercise among Nepalese type 2 diabetic patients. Int Scholarly Res Notices. (2017) 2017:1–9. doi: 10.1155/2017/1273084, PMID: 29349287 PMC5733940

[54] Sohal T Sohal P King-Shier KM Khan NA. Barriers and facilitators for type-2 diabetes management in south Asians: a systematic review. PLoS One. (2015) 10:e0136202. doi: 10.1371/journal.pone.0136202, PMID: 26383535 PMC4575130

[55] Tariq O Rosten C Huber J. Cultural influences on making nutritional adjustments in type 2 diabetes in Pakistan: the perspectives of people living with diabetes and their family members. Qual Health Res. (2024) 34:562–78. doi: 10.1177/10497323231219392, PMID: 38131164

[56] Tran AT Berg TJ Gjelsvik B Mdala I Thue G Cooper JG. Ethnic and gender differences in the management of type 2 diabetes: a cross-sectional study from Norwegian general practice. BMC Health Serv Res. (2019) 19:1–11. doi: 10.1186/s12913-019-4557-4, PMID: 31779621 PMC6883677

[57] Ansari RM Harris MF Hosseinzadeh H Zwar N. Experiences of diabetes self-management: a focus group study among the middle-aged population of rural Pakistan with type 2 diabetes. Diabetology. (2022) 3:17–29. doi: 10.3390/diabetology3010002

[58] Kadariya S Aro AR. Barriers and facilitators to physical activity among urban residents with diabetes in Nepal. PLoS One. (2018) 13:e0199329. doi: 10.1371/journal.pone.0199329, PMID: 29953475 PMC6023206

[59] Byers D. Facilitators and barriers to type 2 diabetes self-management among rural African American adults. J Health Disparities Res Prac. (2016) 9:9.

[60] Parajuli J Saleh F Thapa N Ali L. Factors associated with nonadherence to diet and physical activity among Nepalese type 2 diabetes patients; a cross sectional study. BMC Res Notes. (2014) 7:1–9. doi: 10.1186/1756-0500-7-758, PMID: 25344089 PMC4230343

[61] Hawkins JM. Type 2 diabetes self-management in non-Hispanic black men: a current state of the literature. Curr Diab Rep. (2019) 19:1–6. doi: 10.1007/s11892-019-1131-8, PMID: 30741346

[62] Metta E Haisma H Kessy F Geubbels E Hutter I Bailey A. “It is the medicines that keep us alive”: lived experiences of diabetes medication use and continuity among adults in southeastern Tanzania. BMC Health Serv Res. (2015) 15:1–8. doi: 10.1186/s12913-015-0768-5 25890162 PMC4364569

[63] Shen H Edwards H Courtney M McDowell J Wei J. Barriers and facilitators to diabetes self-management: perspectives of older community dwellers and health professionals in C hina. Int J Nurs Pract. (2013) 19:627–35. doi: 10.1111/ijn.12114, PMID: 24330214

[64] Basu S Garg S. The barriers and challenges toward addressing the social and cultural factors influencing diabetes self-management in Indian populations. J Social Health and Diabetes. (2017) 5:071–6. doi: 10.1055/s-0038-1676245, PMID: 39691772

[65] Bhandari P Kim M. Self-care behaviors of nepalese adults with type 2 diabetes: a mixed methods analysis. Nurs Res. (2016) 65:202–14. doi: 10.1097/NNR.0000000000000153, PMID: 27124256

[66] Wilken M Nunn M. Talking circles to improve diabetes self-care management. Diabetes Educ. (2017) 43:388–95. doi: 10.1177/0145721717706765, PMID: 28494697

[67] Pfeiffer J Li H Martez M Gillespie T. The role of religious behavior in health self-management: a community-based participatory research study. Religion. (2018) 9:357. doi: 10.3390/rel9110357

[68] Onyishi CN Eseadi C Ilechukwu LC Okoro KN Okolie CN Egbule E. Potential influences of religiosity and religious coping strategies on people with diabetes. World J Clin Cases. (2022) 10:8816–26. doi: 10.12998/wjcc.v10.i25.8816, PMID: 36157650 PMC9477035

[69] Amadi K. Religion, coping and outcome in out-patients with depression or diabetes mellitus. Acta Psychiatr Scand. (2016) 133:489–96. doi: 10.1111/acps.12537, PMID: 26667095

[70] Maselko J Hayward RD Hanlon A Buka S Meador K. Religious service attendance and major depression: a case of reverse causality? Am J Epidemiol. (2012) 175:576–83. doi: 10.1093/aje/kwr349, PMID: 22350581 PMC3299417

[71] Weber SR Pargament KI. The role of religion and spirituality in mental health. Curr Opin Psychiatry. (2014) 27:358–63. doi: 10.1097/YCO.0000000000000080, PMID: 25046080

[72] Kato A Fujimaki Y Fujimori S Isogawa A Onishi Y Suzuki R. How self-stigma affects patient activation in persons with type 2 diabetes: a cross-sectional study. BMJ Open. (2020) 10:e034757. doi: 10.1136/bmjopen-2019-034757, PMID: 32423931 PMC7239528

[73] Talukder A Sara SS Hossain MT Nath CD Rahman R Hussain S. Rural and urban differences in the prevalence and determinants of Type-2 diabetes in Bangladesh. PLoS One. (2024) 19:e0298071. doi: 10.1371/journal.pone.0298071, PMID: 38603719 PMC11008877

[74] Syed ST Gerber BS Sharp LK. Traveling towards disease: transportation barriers to health care access. J Community Health. (2013) 38:976–93. doi: 10.1007/s10900-013-9681-1, PMID: 23543372 PMC4265215

[75] Ilag BN. Investigating a quantitative study on the opportunities and challenges in implementing hybrid-remote work environment of telehealth in the US rural populations. Northcentral University ProQuest Dissertations & Theses (2023).

[76] Sarker AR Sultana M Alam K Ali N Sheikh N Akram R. Households' out-of-pocket expenditure for healthcare in Bangladesh: a health financing incidence analysis. Int J Health Plann Manag. (2021) 36:2106–17. doi: 10.1002/hpm.3275, PMID: 34218437

[77] Ide N LoGerfo JP Karmacharya B. Barriers and facilitators of diabetes services in Nepal: a qualitative evaluation. Health Policy Plan. (2018) 33:474–82. doi: 10.1093/heapol/czy011, PMID: 29447397

[78] Ghammari F Jalilian H Khodayari-zarnaq R Gholizadeh M. Barriers and facilitators to type 2 diabetes management among slum-dwellers: a systematic review and qualitative meta-synthesis. Health Sci Reports. (2023) 6:e1231. doi: 10.1002/hsr2.1231, PMID: 37123550 PMC10140644

[79] Wang Y Hu XJ Wang HHX Duan HY Chen Y Li YT. Follow-up care delivery in community-based hypertension and type 2 diabetes management: a multi-Centre, survey study among rural primary care physicians in China. BMC Fam Pract. (2021) 22:1–10. doi: 10.1186/s12875-021-01564-z, PMID: 34774003 PMC8590343

[80] Chowdhury HA Harrison CL Siddiquea BN Tissera S Afroz A Ali L. The effectiveness of diabetes self-management education intervention on glycaemic control and cardiometabolic risk in adults with type 2 diabetes in low-and middle-income countries: a systematic review and meta-analysis. PLoS One. (2024) 19:e0297328. doi: 10.1371/journal.pone.0297328, PMID: 38306363 PMC10836683

[81] Attridge M Creamer J Ramsden M Cannings-John R Hawthorne K. Culturally appropriate health education for people in ethnic minority groups with type 2 diabetes mellitus. Cochrane Database Syst Rev. (2014) 2014:CD006424. doi: 10.1002/14651858.CD006424.pub3, PMID: 25188210 PMC10680058

[82] Lepard MG Joseph AL Agne AA Cherrington AL. Diabetes self-management interventions for adults with type 2 diabetes living in rural areas: a systematic literature review. Curr Diab Rep. (2015) 15:1–12. doi: 10.1007/s11892-015-0608-3, PMID: 25948497 PMC5373659

[83] Szczepura A. Access to health care for ethnic minority populations. Postgrad Med J. (2005) 81:141–7. doi: 10.1136/pgmj.2004.026237, PMID: 15749788 PMC1743229

