# Supplementary material for: Factors influencing type 2 diabetes self-management practices in rural Bangladesh: a qualitative investigation
Source: Front Public Health. 2025 Jan 15;12:1508204. doi: 10.3389/fpubh.2024.1508204 (PMC11774903; doi:10.3389/fpubh.2024.1508204)
Supplement: Supplementary file 1 [file Data_Sheet_1.docx]

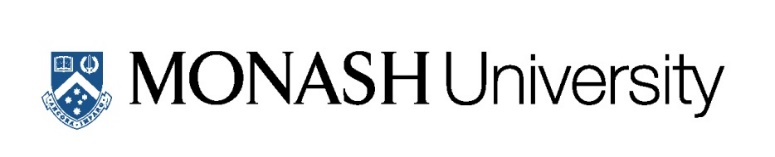


**Focus Group Discussion Guide: People with Type 2 Diabetes Mellitus**

Greetings!! My name is _________ and I am calling on behalf of Monash University. Thank you for volunteering for this focus group.

As discussed before,

- Today, we would like to hear about your experiences with type 2 diabetes self-management and I'll ask you about the challenges you face and the factors that assist you in managing your diabetes
- We also want to hear your expectation of the necessity of an educational program on self-management of diabetes care at the community level.
- The focus group discussion will take no more than one hour.
- Are you still happy to part-take in this research?

To ensure all of the information you provide in the interview is interpreted accurately by us, we would like to audio-tape the interview. The tapes and information provided will remain confidential and you will not be identified by name during the analysis nor details provided about your position that could identify you as an individual during the analysis and reporting. You are requested to try to answer and comment as accurately and truthfully as possible. I and the other focus group participants would appreciate it if you would refrain from discussing the comments of other group members outside the focus group. If there are any questions or discussions that you do not wish to answer or participate in, you do not have to do so; however please try to answer and be as involved as possible.

Are you happy to consent to the interview being recorded?

**Ground rules**

- The most important rule is that only one person speaks at a time. There may be a temptation to join in when someone is talking, but please wait until they have finished.
- There are no right or wrong answers
- You do not have to speak in any particular order
- When you do have something to say, please do so. There are many of you in the group and it is important that I obtain the views of each of you
- You do not have to agree with the views of other people in the group
- Does anyone have any questions? (answers).

Okay, let’s start

Date………………..

No of participants……………

**Warm-up**

First, we would like everyone to introduce themselves. Can you please tell us your name?

**Guiding questions for Focus Group Discussion**

| **Sl no** | **Topic** | **Probes** |
| --- | --- | --- |
| **1**  **Knowledge** | How do you manage your diabetes?  Please elaborately discuss | - Eating a healthy diet - Being physically active - Test blood sugar regularly - Taking medicines as prescribed - Learning ways to manage stress - Coping with the emotional side of diabetes - Going to checkups regularly - Anything else |
| **2**  **Motivation** | What is the most important issue to you when you think about managing your diabetes (i.e. what motivates you the most)  Is anyone happy to share his or her experience?  Please elaborately discuss | - Better health outcomes - Long-term health outcome/avoid diabetes-related complications - Being healthy for themselves/family - Reduce the cost of diabetes care - Anything else |
| **Facilitators of diabetes self-management practices** | | |
| **3**  **Facilitators** | What are some of the things you are doing now that you feel positively contribute to managing your diabetes? | • Diet  Exercise  Adhere to taking medicine  Regular monitoring of blood glucose  Sleep  • Stress management  Anything else |
| **4** | When you think about managing your diabetes, what or who makes this easier for you and why?  Please elaborately describe how, and why | Patient itself  Family (e.g. family awareness, financial support, help in blood glucose monitoring/ taking medicine/cooking/accompanying to doctor and hospital visits)  Friends/neighbour (e.g. accompanying to doctor/hospital visits)  Work arrangement   - Geographical access to the healthcare center   Healthcare service professional interaction and support   - **Community support** (e.g. cultural values, availability and accessibility of resources, or anything else) - **Government support** (e.g. patient-friendly guidelines, funding for free medicine and blood glucose monitoring kits/devices) - **Anything else** |
| **5** | What role do you believe your local hospital/healthcare provider plays in your diabetes?  Please elaborately describe how, and why | - Focusing on patient-centered care - Build trusting/friendly relationships - Provide emotional support - Provide access to information - Utilization of resources (i.e. staff, guidelines, equipment, and referral system) - Minimise cost of care - Anything else |
| **Barriers to diabetes self-management practices** | | |
| **6** | What things make it more difficult to manage your diabetes?  Please elaborately describe how and why? | Family (e.g. family awareness, financial support, help in blood glucose monitoring/ taking medicine/cooking/accompanying to doctor and hospital visits)  Friends/neighbours (e.g. lack of supportive attitude)  Work arrangement  Geographical access to the healthcare center  Healthcare service professional interaction and support   - Community support (e.g. cultural values, availability and accessibility of resources, or anything else) - Government support (e.g. patient-friendly guidelines, funding for free medicine and blood glucose monitoring kits/devices) - Any thing else |
| **7** | Were you able to overcome these barriers, and if so how?  Please elaborately describe | - Practicing self-management/healthy lifestyle on a regular basis |
| **8** | What challenges do you face in managing your diabetes within the local hospital/healthcare providers/diabetes hospital?  Please elaborately describe what, when, how, and why | - Access to services - Access to information - Utilization of resources (i.e. staff, guidelines, equipment, and referral system) - Cost of diabetes care - Patient-medical staff relationship - Anything else |
| **Expectation, implementation, and adoption capacity** | | |
| **9** | Who do you think could be the best people to support you in managing your diabetes how, and, why?  Please elaborately describe | • Family  • Friends/neighbours  • Healthcare service providers  • Community members   - Anything else |
| **10** | What do you believe would help you to improve your skills/knowledge in diabetes management if it are offered to you?    Please elaborately describe | More health information to improve knowledge  More personalised support  Easy access to support if I need it  Someone to help me manage my diabetes, for example, a health coach—set goals, plans, feedback, and accountability  Strategies, ideas  Locally accessible resources/programs  Better blood glucose monitoring devices  Anything else |
| **11** | Where would you prefer to attend this service?  Please elaborately describe | • Local hospital setting (e.g. community clinic, upazila health complex)  • Community setting (e.g.local primary /high school, religious centers)  • Local informal healthcare setting (e.g. unqualified practitioners and homeopaths’ place, local club room or yard)  • Allied health setting (e.g. dietitian, nutritionist)  • Online   - Anything else |
| **12** | How would you prefer to attend this service? | In a group or individual delivery format?  With an intervention manual (e.g. text/pictorial materials)?  With ongoing support via regular phone calls or home visits?  With your family members or not?  Delivered by who?  Who would motivate them to attend? If anyone describes elaborately |
| **13** | How intensely would you like to be supported? | - Duration of the program - Frequency of the program - Self-guided |
| **14** | How willing are you to pay for this service? | - Would paying help motivate you? |

**Conclusion**

- Thank you for participating. This has been a very successful discussion
- Your opinions will be a valuable asset to the study
- We hope you have found the discussion interesting


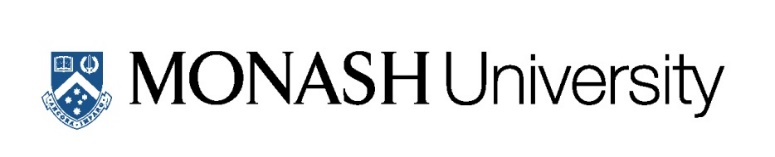


**Focus Group Discussion Guide: Caregivers**

Greetings! My name is ___________ and I am calling on behalf of Monash University. As discussed previously,

- Today I would like to hear your opinions on the problems/challenges/issues and helping factors experienced by people with type 2 diabetes to perform their self-management of diabetes.
- I also want to hear your expectation of the necessity of an educational program on self-management of diabetes care at the community level.
- The focus group discussion will take no more than one hour.
- Are you still happy to part-take in this research?

To ensure all of the information you provide in the interview is interpreted accurately by us, we would like to audio-tape the interview. The tapes and information provided will remain confidential and you will not be identified by name during the analysis nor details provided about your position that could identify you as an individual during the analysis and reporting. You are requested to try to answer and comment as accurately and truthfully as possible. I and the other focus group participants would appreciate it if you would refrain from discussing the comments of other group members outside the focus group. If there are any questions or discussions that you do not wish to answer or participate in, you do not have to do so; however please try to answer and be as involved as possible.

Are you happy to consent to the interview being recorded?

**Ground rules**

- The most important rule is that only one person speaks at a time. There may be a temptation to join in when someone is talking, but please wait until they have finished.
- There are no right or wrong answers
- You do not have to speak in any particular order
- When you do have something to say, please do so. There are many of you in the group and it is important that I obtain the views of each of you
- You do not have to agree with the views of other people in the group
- Does anyone have any questions? (answers).

Okay, let’s start

Date………………..

No of participants……………

**Warm-up**

First, we would like everyone to introduce themselves. Can you please tell us your name?

**Guiding questions for Focus Group Discussion (Caregivers)**

| **Sl no** | **Topic** | **Probes** |
| --- | --- | --- |
| **1** | In your family, how does the person you provide care for/support manage their diabetes?  Please elaborately discuss | - Eat a healthy diet - Being physically active - Test blood sugar regularly - Take medicines as prescribed - Learn ways to manage stress - Cope with the emotional side of diabetes - Go to checkups regularly - Anything else |
| **2** | What is most important to you when you think about assisting or supporting people with type 2 diabetes (i.e. what motivates you the most)  Is anyone happy to share his or her experience?  Please elaborately discuss | - Better health outcomes - Long-term health outcomes/avoid diabetes-related complications - Being healthy for themselves/family - Reduce the cost of diabetes care - Anything else |
| **Facilitators of diabetes self-management practices** | | |
| **3** | What are some of the things you are doing now as a caregiver that you feel positively contribute to managing their diabetes?  Please elaborately describe how, and why | • Diet  Exercise  Adhere to taking medicine  Help with regular monitoring of blood glucose  Accompanying doctor and hospital visits  Financial support  Cooking foods  Sleep management  • Stress management  Anything else |
| **4** | When thinking about managing diabetes, what or who makes this easier for people with type 2 diabetes and why?  Please elaborately describe | Patient itself (e.g. knowledge, motivation, responsibility, or anything else)  Work arrangement  Geographical access to the healthcare center/facilities  Healthcare service professional interaction and support   - Community support (e.g. cultural values, availability and accessibility of resources, or anything else) - Government support (e.g. patient-friendly guidelines, funding for free medicine and blood glucose monitoring kits/devices) |
| **5** | What role do you believe your local hospital/healthcare provider plays in diabetes?  Please elaborately describe how, and why | - Focusing on patient-centered care - Build trusting/friendly relationships - Provide emotional support - Provide access to information - Utilization of resources (i.e. staff, guidelines, equipment, and referral system) - Minimise cost of care - Anything else |
| **Barriers to diabetes self-management practices** | | |
| **6** | What things make it more difficult for your patients to manage their diabetes?  Please elaborately describe | Patient itself  Family (e.g. lack of family support)  Friends/neighbours  Work arrangement  Geographical access to the healthcare center  Healthcare service professional interaction and support(i.e. staff, guidelines, equipment, and referral system)   - Community support - Government support(e.g. patient-friendly guidelines, funding for free medicine and blood glucose monitoring kits/devices) |
| **7** | Have they been able to overcome these barriers, and if so how?  Please elaborately describe | - Practicing self-management/healthy lifestyle on a regular basis |
| **8** | What challenges do they face in managing their diabetes within the local hospital/healthcare providers?  Please elaborately describe what, when, how, and why | - Access to services - Access to information - Utilization of resources (i.e. staff, guidelines, equipment, and referral system) - Cost of diabetes care, or - Anything else |
| **Expectation, implementation, and adoption** | | |
| **9** | Who do you think would be the best people to support people with type 2 diabetes in managing their diabetes and why?  Please elaborately describe | • Family  • Friends/neighbours  • Healthcare service providers  • Community members (chairman, member, school teacher, college teacher) |
| **10** | What do you believe would help people with type 2 diabetes to improve their skills/knowledge in diabetes management if it are offered to them?  Please elaborately describe | More health information to improve knowledge  More personalised support  Easy access to support if they need it  Someone to help people with type 2 diabetes to manage their diabetes, for example, a health coach—set goals, plans, feedback, and accountability  Strategies, ideas  Locally accessible resources/programs  Availability of blood glucose monitoring kits/devices |
| **11** | Where do you think would be an ideal setting to attend a service like this? | • Local hospital setting (e.g. community clinic, upazila health complex)  • Community setting (e.g.local primary /high school, religious centers)  • Local informal healthcare setting (e.g. unqualified practitioners and homeopaths’ place, local club room, yard)  • Allied health setting (eg dietitian, nutritionist)  • Online  Anything else |
| **12** | How would they prefer to attend this service? | In a group or individual delivery format?  Intervention manual (text/pictorial materials)  With ongoing support via regular phone calls or home visits?  With family members or not?  Delivered by who  Who would motivate them to attend? If anyone |
| **Conclusions** | | |
| - Thank you for participating. This has been a very successful discussion - Your opinions will be a valuable asset to the study - We hope you have found the discussion interesting | | |
